# Notice of Plagiarism and Copyright Violation

**DOI:** 10.4269/ajtmh.2012.01-025.notice

**Published:** 2012-08-01

It has been brought to our attention that the following article:

Haggag SH, Abdullah SM, 2011. Molecular diagnosis of *Schistosoma mansoni* infection in human serum and feces by using polymerase chain reaction. *Life Science Journal* *8(2):* 398–404. http://www.lifesciencesite.com/lsj/life0802/54_5171life0802_398_404.pdf

is nearly identical to an article published in the *American Journal of Tropical Medicine and Hygiene*:

Pontes LA, Dias-Neto E, Rabello A, 2002. Detection by polymerase chain reaction of *Schistosoma mansoni* DNA in human serum and feces. *Am J Trop Med Hyg 66(2):* 157–162. http://www.ajtmh.org/content/66/2/157.full.pdf+html

While we acknowledge some differences in the Materials and Methods, including the number of samples, kit type, and apparatus type, the text is nearly identical throughout the rest of paper, particularly in the introduction and discussion. The references are nearly identical as well.

After receiving no response from the authors, the editor, or the publisher, we feel compelled to publish an announcement in our journal denouncing this work.

